# Regulation of PKM2 and Nrf2-ARE Pathway during Benzoquinone Induced Oxidative Stress in Yolk Sac Hematopoietic Stem Cells

**DOI:** 10.1371/journal.pone.0113733

**Published:** 2014-12-01

**Authors:** Jie Zhu, Zhuoyue Bi, Tan Yang, Wei Wang, Zhen Li, Wenting Huang, Liping Wang, Shaozun Zhang, Yanfeng Zhou, Ningna Fan, YuE Bai, Wentao Song, Chunhong Wang, Hong Wang, Yongyi Bi

**Affiliations:** 1 School of Public Health, Wuhan University, Wuhan, Hubei, P.R. China; 2 Hubei Key Laboratory of Allergy and Immune-related Diseases, Wuhan, Hubei, P.R. China; 3 Hubei Biomass-resource Chemistry and Environmental Biotechnology Key Laboratory, Wuhan University, Wuhan, Hubei, P.R. China; 4 Hubei Provincial Key Laboratory for Applied Toxicology (Hubei Provincial Academy for Preventive Medicine), Wuhan, P.R. China; 5 School of Public Health, Kunming Medical University, Chenggong District, Kunming, P.R. China; 6 Nanchang Center for Disease Control and Prevention, Nanchang, P.R. China; The University of Texas M.D Anderson Cancer Center, United States of America

## Abstract

Benzene is an occupational toxicant and an environmental pollutant that is able to induce the production of reactive oxygen species (ROS), causing oxidative stress and damages of the macromolecules in target cells, such as the hematopoietic stem cells. We had previously found that embryonic yolk sac hematopoietic stem cells (YS-HSCs) are more sensitive to benzene toxicity than the adult bone marrow hematopoietic stem cells, and that nuclear factor-erythroid-2-related factor 2 (Nrf2) is the major regulator of cytoprotective responses to oxidative stress. In the present report, we investigated the effect of PKM2 and Nrf2-ARE pathway on the cellular antioxidant response to oxidative stress induced by benzene metabolite benzoquinone (BQ) in YS-HSC isolated from embryonic yolk sac and enriched by magnetic-activated cell sorting (MACS). Treatment of the YS-HSC with various concentrations of BQ for 6 hours induces ROS generation in a dose-dependent manner. Additional tests showed that BQ is also capable of inducing expression of NADPH oxidase1 (NOX1), and several other antioxidant enzymes or drug-metabolizing enzymes, including heme oxygenase 1 (HMOX1), superoxide dismutase (SOD), catalase and NAD(P)H dehydrogenase quinone 1 (NQO1). Concomitantly, only the expression of PKM2 protein was decreased by the treatment of BQ but not the PKM2 mRNA, which suggested that BQ may induce PKM2 degradation. Pretreatment of the cells with antioxidant N-acetylcysteine (NAC) decreased ROS generation and prevented BQ-induced PKM2 degradation, suggesting involvement of ROS in the PKM2 protein degradation in cellular response to BQ. These findings suggest that BQ is a potent inducer of ROS generation and the subsequent antioxidant responses of the YS-HSC. The accumulated ROS may attenuate the expression of PKM2, a key regulator of the pyruvate metabolism and glycolysis.

## Introduction

Benzene is a known human leukemogen. Epidemiological studies revealed that occupational or environmental exposure to benzene during pregnancy is associated with increased risk for the development of childhood malignancies, such as leukemia [1]–[7]. Experiments using animal models showed an increased frequency of chromatid breaks and DNA recombination exchange in fetal hematopoietic cells following benzene treatment [8], [9]. Despite extensive studies in the past years, relatively little is known about the detailed carcinogenic mechanism of benzene. Accumulating evidence suggests that one of the underlying mechanisms of benzene-induced carcinogenesity is oxidative damage caused by its metabolites.

Benzene metabolized primarily in the liver involving cytochromes P450-catalyzed oxidation of benzene to benzene oxide by addition of oxygen atom to the benzene ring. Benzene oxide then further biotransformed to phenol (PH), hydroquinone (HQ) and 1,4-benzoquinone (BQ). BQ is the most potent benzene metabolites in inducing generation of the reactive oxygen species (ROS), such as hydrogen peroxide (H_2_O_2_), superoxide anion radicals (O2.-) and hydroxyl radicals (HO.) [10]. The increased ROS are able to cause oxidative damage of the cellular macromolecules in the target cells [11]–[13]. Although several biological sources have been linked to ROS generation, the most important source of ROS in cellular response to BQ is the NADPH oxidase 1 [14]. ROS are highly capable of disrupting normal hematopoiesis, which may attribute to the utero-initiated benzene toxicity [9], [15], [16].

A variety of redox systems and enzymatic mechanisms have been shown to be protective against ROS-induced oxidative stress responses. The nuclear factor-erythroid-2-related factor 2 (Nrf2) is known as a critical protective factor in preventing cellular or tissue damage caused by ROS or xenobiotics, such as benzene metabolite BQ [17]–[19]. It has been well-established that Nrf2 is directly associated with Keap1 in the cytoplasm under normal condition [20]. Elevation of ROS induces nuclear accumulation of Nrf2 and the activation of Nrf2-ARE pathway, including antioxidant genes, phase II detoxifying enzymes and other related proteins, such as catalase, superoxide dismutase (SOD), NAD(P)H: quinone oxidoreductase-1(NQO1), heme oxygenase-1 (HMOX1) [17], [21]. Pyruvate kinase M2 (PKM2) is a major glycolytic enzyme for glucose metabolism in cancer cells [22]. A recent study showed that acute ROS accumulation caused inhibition of pyruvate kinase M2 (PKM2), spontaneously transfer glucose flux into the pentose phosphate pathway and thereby generate sufficient anti-oxidant responses for the detoxification of ROS [23].

We had previously established an experimental system for assessment of the hematopoietic toxicity and leukemogenicity of benzene and its metabolites during mouse embryonic development. We found that the cytotoxic and apoptotic effects of benzene metabolites were much pronounced in embryonic YS-HSCs than in adult BM-HSCs [24]. The present study was focused on the possible involvement of ROS generation and the activation of the anti-oxidant defense system in embryonic YS-HSCs treated with BQ. We provided evidence showing that BQ is highly capable of inducing ROS, along with an increased elevation of NOX1 protein, nuclear accumulation of Nrf2 and the activation of Nrf2-ARE pathway in YS-HSCs. The expression of PKM2 protein was decreased by treatment of BQ. Inhibition of PKM2 may play an important role in determining the biological effects of benzene-induced ROS in yolk sac hematopoietic cells. Pretreatment of YS-HSCs with antioxidant NAC reversed PKM2 reduction, suggesting BQ-induced ROS generation contributed to PKM2 degradation.

## Materials and Methods

### Ethics Statement

As described in detail previously [24], all experiments were approved by the Institutional Animal Care and Use Committee of Wuhan University (No. 10061), and the animal procedures were performed in accordance with institutional and national guidelines. All surgery was performed under anesthesia with ketamine and lidocaine, and all efforts were made to minimize suffering.

### Embryonic YS-HSCs isolation and proliferation

Special pathogen free (SPF) Kunming mice were purchased from the Central Animal Facility of Wuhan University as described previously [24]. Kunming mice were originated from Swiss mice and were brought to Kunming, China, from the Indian Haffkine Institute in 1944. This mice line has been widely utilized in pharmacological, toxicological, medicinal and biological research and testing because of its' high disease resistance, good adaptive capacity, high breeding coefficient, and good survival rate.

Isolation of yolk sac cells from a pregnant female on 8–10 days post conception were performed according to previous report [24]. Briefly, the yolk sacs were separated from each embryo conceptus by removing the endometrial tissues, placental tissues and their junctures. The tissues were pelleted by a brief spin and were resuspended in 0.25% collagenase in PBS with 20% FBS, incubated at 37°C for 40 min. After incubation, the cell pellets were dissociated into single cell suspensions by gently repeat pipetting.

After separation of yolk sac cells, the cells were analyzed for surface expression of hematopoietic markers. Cells were washed and fluorescently stained with mouse CD117 antibodies conjugated to PE (#130-0991-730), CD41-FITC or CD45-FITC (eBioscience, San Diego, CA, USA). Cells were incubated for 10 minutes in the dark on ice. Finally, cells were washed and resuspended in phosphate buffered saline (PBS) containing 0.5% bovine serum albumin (BSA). Flow cytometric analysis was carried out with a flow cytometer (Epics Altra II, Beckman, USA).

As described in detail previously [24], the yolk cells was first depleted of lineage positive (Lin^+^) cells using magnetic columns and was then positively selected by using anti-murine CD117 antibodies conjugated to mini-magnetic beads (Miltenyi Biotec, Inc., Auburn, CA, http://www.miltenyibiotec.com ). Up to 3.2×10^6^ of lineage^−^ CD117^+^ cells were isolated from eight yolk sac cell suspensions. Isolated YS-HSC cells were cultured at 37°C with 5% humidified CO_2_ in the Serum-Free Expansion Medium (Stem Cell Technologies, Inc, Vancouver, BC, Canada) with the addition of cytokines SCF (100 ng/ml), IL-3 (20 ng/ml), and Flt-3 (100 ng/ml) (Peprotech USA). Cell were grown in polystyrene 6-well plates in a volume of 2 ml per well up to 0.6×10^6^ cells/ml and kept in fresh culture medium for 12 h before treatments.

### Analysis of intracellular ROS

To assess the generation of ROS in YS-HSCs cells, freshly isolated YS-HSCs cells (5×10^5^/ml) were incubated for 6 hours with 10 µM and 20 µM concentrations of BQ with or without 2 hours pretreatment of N-acetylcysteine (Sigma). Samples of the cultures were loaded with 20 µM DCF-DA (Sigma) and incubated at 37°C for 45 min. The peak excitation wavelength for oxidized DCF was 488 nm. We used a flow cytometer (Epics Altra II, Beckman , USA) to measure ROS generation by the fluorescence intensity. Quantitative assay of ROS generation was performed by normalization to the control group. For fluorescence microscopic analysis, the cells were washed with PBS, mounted, and observed under an inverted phase-contrast and fluorescence microscope. (OLYMPUS IX71, Japan).

### Nuclear and cytoplasmic extraction

Cells were seeded in 6-well plates at density of 3×10^6^/well and treated with 0, 5, 10, 20, 40 µ M BQ for 6 hours. After treatment, nuclear and cytoplasmic proteins were extracted using a Nuclear and Cytoplasmic Protein Extraction Kit (Beyotime Biotechnology) according to the manufacturer's recommendations. Cells were washed twice with ice-cold PBS. Cell suspension was made by addiing 200 µl ice-cold Cytoplasmic Extraction Reagent I (CER I) to the cell pellets, vortexing and incubating the tube on ice for 10 minutes. The Cytoplasmic Extraction Reagent II (CER II, 10 µl) was then added to the tube, vortexed for 5 seconds on the highest setting, incubated on ice for 1 minute and centrifuged (14,000×g) the tube for 5 minutes at 4°C. The supernatant (cytoplasmic extract) was collected to a clean pre-chilled tube. The insoluble was suspended in 50 µl Nuclear Extraction Reagent and incubated on ice for a total of 40 minutes, vortexing for 15 seconds every 10 minutes and centrifuged the tube for 10 minutes at 4°C. The supernatant (nuclear extract) was collected. The extracts were stored at −80°C until use.

### Whole-cell protein extraction

Cells (3.0−3.5×10^6^/ml) were washed twice with ice-cold PBS and then lysed in a lysis buffer (2 mM sodium orthovanadate, 50 mM NaF, 20 mM HEPES, pH 7.5, 150 mM NaCl, 1.5 mM MgCl_2_, 5 mM sodium pyrophosphate, 10% glycerol, 0.2%Triton X-100, 5 mM EDTA, 1 mM PMSF, 10 mg/ml leupeptin, and 10 mg/ml aprotinin) on ice for 30 min. Insoluble materials were pelleted at 14,000×g for 20 min at 4°C. The supernatant (whole-cell extract) was collected and stored at −80°C until use.

### Immunoblotting

Cell lysates was mixed with the SDS sample buffer, and was boiled for 5 min. Equal amounts of protein (30 µg) were electrophoresed on 10% SDS/PAGE and were electroblotted onto PVDF membranes and identified with rabbit polyclonal antibody to NOX1, SOD2 (Abcam), SOD1, Catalase, PKM2 (Cell Signaling Technology), HMOX1, NQO1, GAPDH (Bioworld Technology). Secondary goat anti-rabbit IgG–HRP (Bioworld Technology) was used according to the species of primary antibody. Chemiluminescence was performed using SuperSignal West Dura (Pierce) and the FluorChem 8900 visualization system (Alpha Innotech). The relative density of detected signals was analyzed by using the image analysis software (Image 1.56, NIH).

### Statistical analysis

SPSS 17.0 was used for data analysis. Data were expressed as means ± SDs. The statistical analysis was determined by one-way analysis of variance (ANOVA) followed by Least Significant Difference test (LSD) and Dunnett's T3. *P* values less than 0.05 were considered significant.

## Results and Discussion

### Characterization of hematopoietic surface marker expression in E8-10 (embryonic day 8–10) murine yolk sacs

**Figure 1:**
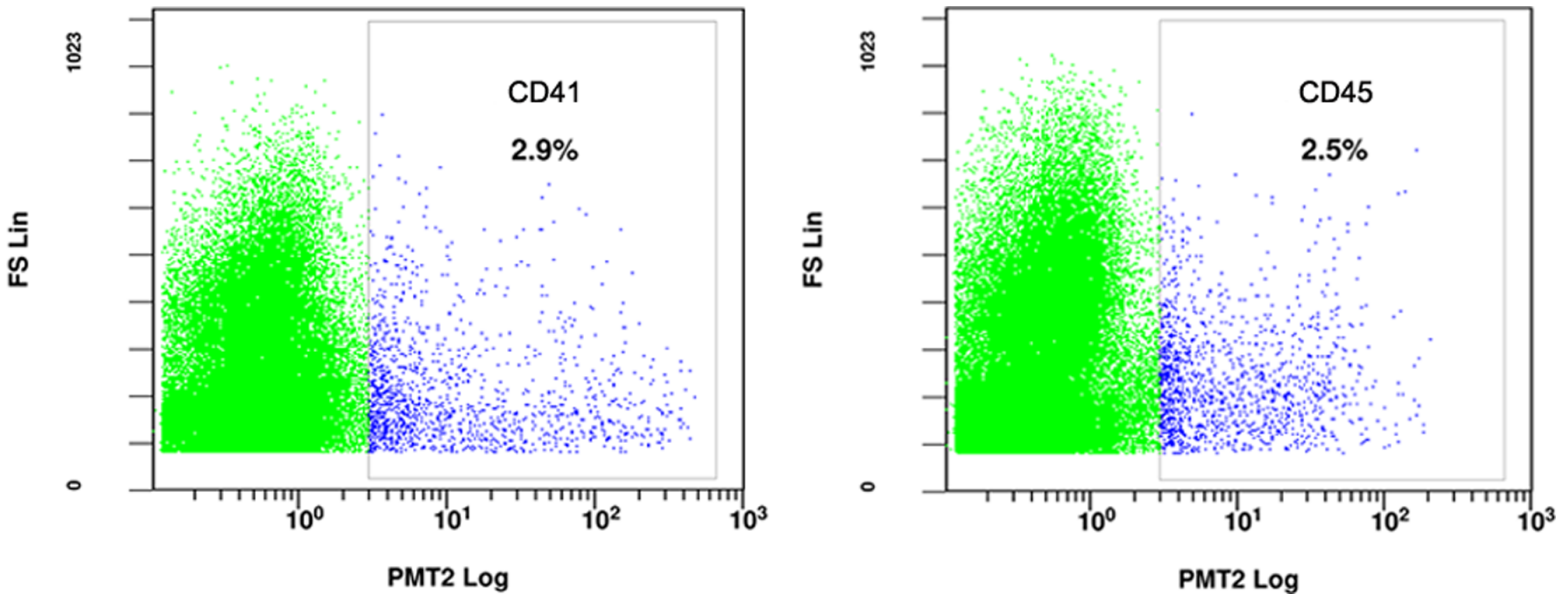
Expression of hematopoietic surface markers in E8-10 (embryonic day 8–10) murine yolk sacs. Representative flow cytometric data from more than three independent analyses were shown.

We had previously found that CD41-expressing cells were present in the E9 murine yolk sacs through immunohistochemical staining [24]. To additionally validate this finding, we stained the yolk sac cells with antibodies against the hematopoietic stem cell antigen markers and quantified the stem cells by flow cytometry (Figure 1, Table 1 and Table S1). The E8-10d yolk sac cells express several HSC markers, including CD117, CD41 and panhematopoietic marker CD45. As described in detail previously, about 19.35±6.29% yolk sac cells express CD117 [24]. Whereas the percentage of CD41- and CD45-positive cells is relatively lower, only about 2.98±1.23% and 2.7±0.44%, respectively.

**Table 1:** Expression of hematopoietic surface makers in E8-10d yolk sac cells.

Maker	Positive cells, %
CD41	2.98±1.23%(n = 11)
CD45	2.7±0.44%(n = 3)

### Exposure of YS-HSCs to BQ increased ROS generation

**Figure 2:**
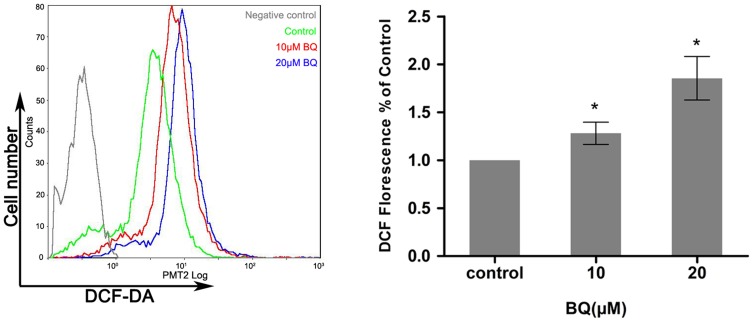
Exposure of YS-HSCs to BQ increased ROS generation. YS-HSCs cells were isolated and treated with 10 µM BQ, 20 µM BQ, or without BQ. The intracellular ROS concentrations were measured by DCF-DA staining. Illustrated overlays on the left panel reflected the ROS generation. Quantifications of ROS generation are displayed on the right pannel. Data are expressed as the mean ± standard deviation (n>3), * *p*<0.01, as compared to the control group.

**Figure 3:**
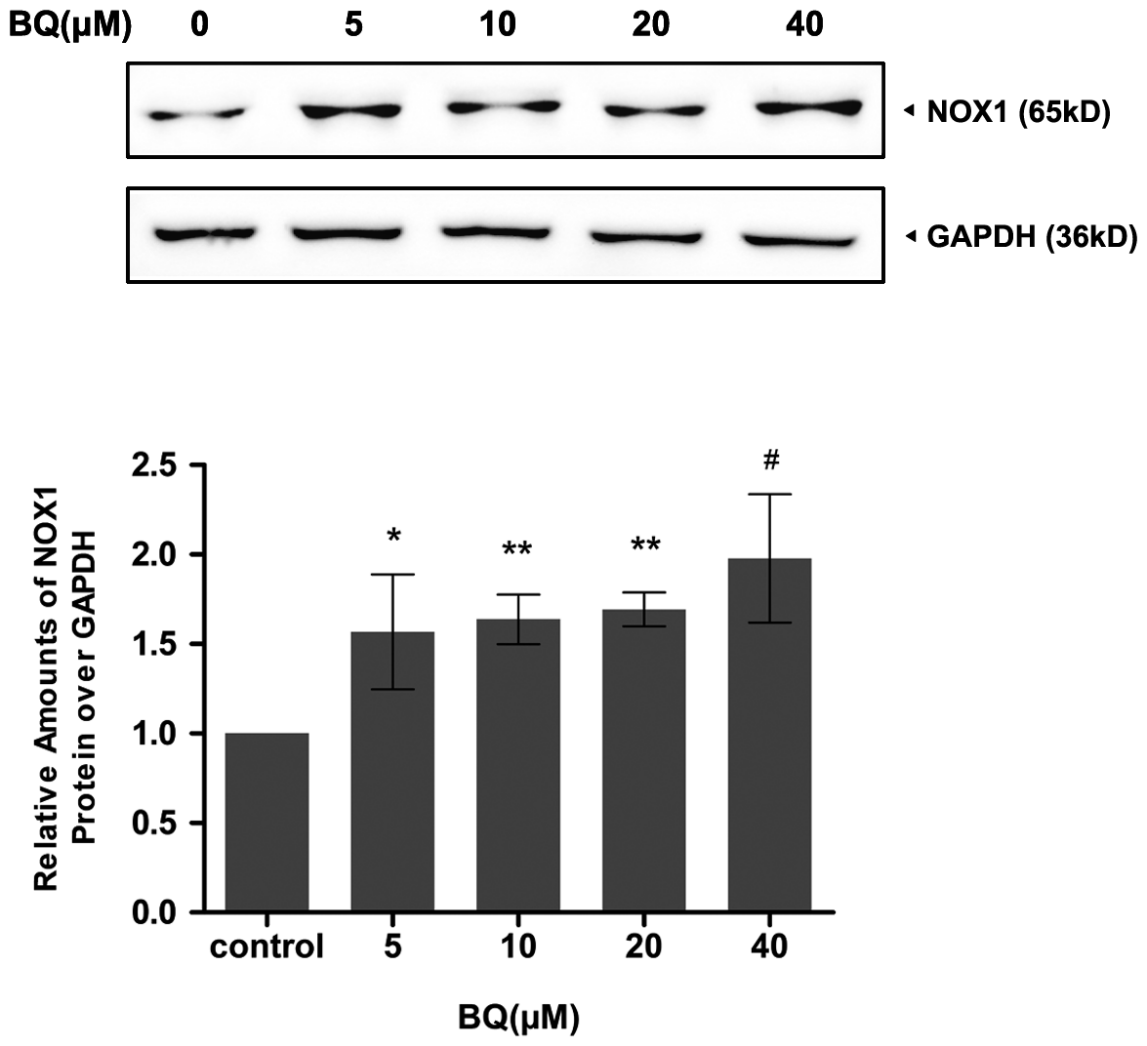
The ROS-generating NADPH oxidase NOX1 was induced by BQ. Immunoblotting data were shown in the upper panel. GAPDH was measured as loading control. Densitometry of immunoblotting results was shown in the lower panel. Values are presented as means ± SDs (n = 3−5). *, *P*<0.05; **, *P*<0.01; #, *P*<0.001 compared with controls.

To examine the capability of BQ in the induction of ROS generation in embryonic primitive hematopoietic cells, YS-HSCs were treated with different concentrations (10 µM, 20 µM) of BQ for 6 hours. ROS generation was examined by flow cytometry and fluorescence microscopy detecting the DCF-DA signals. Levels of ROS were significantly elevated by BQ treatment, as evidenced by right shifted fluorescence intensity (Figure 2). Since intracellular ROS generation is chiefly regulated by NADPH oxidase, the activation status of NADPH oxidase was determined following the treatment of the YS-HSC with BQ. An increased protein level of NOX1 was observed in the cells treated with BQ, suggesting that BQ is a potential activator of NOX1 that is the key subunit of NADPH oxidase linked to ROS generation (Figure 3).

### Degradation of PKM2 by BQ exposure

**Figure 4:**
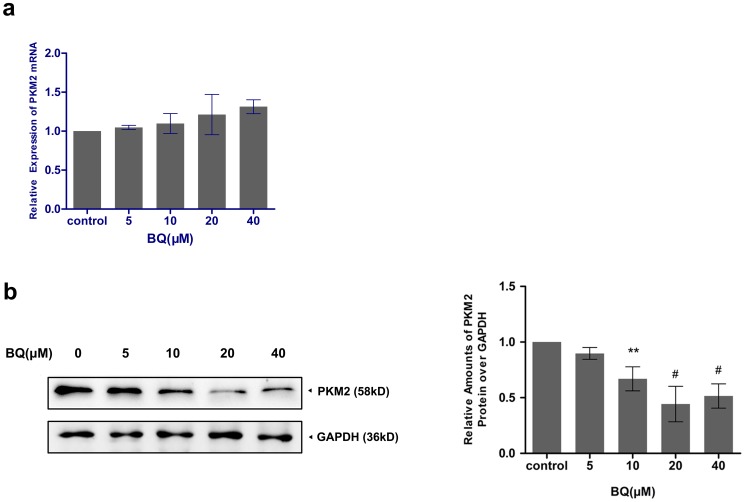
Degradation of PKM2 by BQ exposure. (a)Effect of BQ on PKM2 mRNA expression. Cells were treated with BQ at increasing concentrations and expression of PKM2 mRNA was measured by quantitative real–time PCR. The relative expression of target genes was calculated using the 2-ΔΔCt method. Data represent means ± SDs of at least three individual experiments. (b)The expression of PKM2 protein was decreased by treatment of BQ. Immunoblotting of PKM2 was shown in the left panel. GAPDH was measured as loading control. Densitometry of immunoblot results was shown in the right panel. Values are presented as means ± SDs (n = 3−5). *, *P*<0.05; **P<0.01; #, *P*<0.001 compared with controls.

To examine the effect of BQ on PKM2 expression in YS-HSC, the mRNA levels and protein levels of PKM2 were determined by quantitative real–time PCR and Western blotting, respectively. PKM2 gene expression levels exhibited no statistically significant differences among control and all BQ treated groups in YS-HSC (Figure 4a). On the other hand, exposure to various concentrations of BQ for 6 h resulted in decreased expression levels of PKM2 proteins. As shown in Figure 4b, BQ reduced the expression of PKM2 protein expression in a concentration-dependent manner in YS-HSC. A notable inhibition PKM2 was observed in the cells treated with 10 to 40 µM BQ (<0.5-fold). BQ only reduced PKM2 protein but not the PKM2 mRNA, suggesting that BQ may induce PKM2 degradation.

### Exposure to BQ upregulated Nrf2 protein expression and caused Nrf2 nuclear accumulation

**Figure 5:**
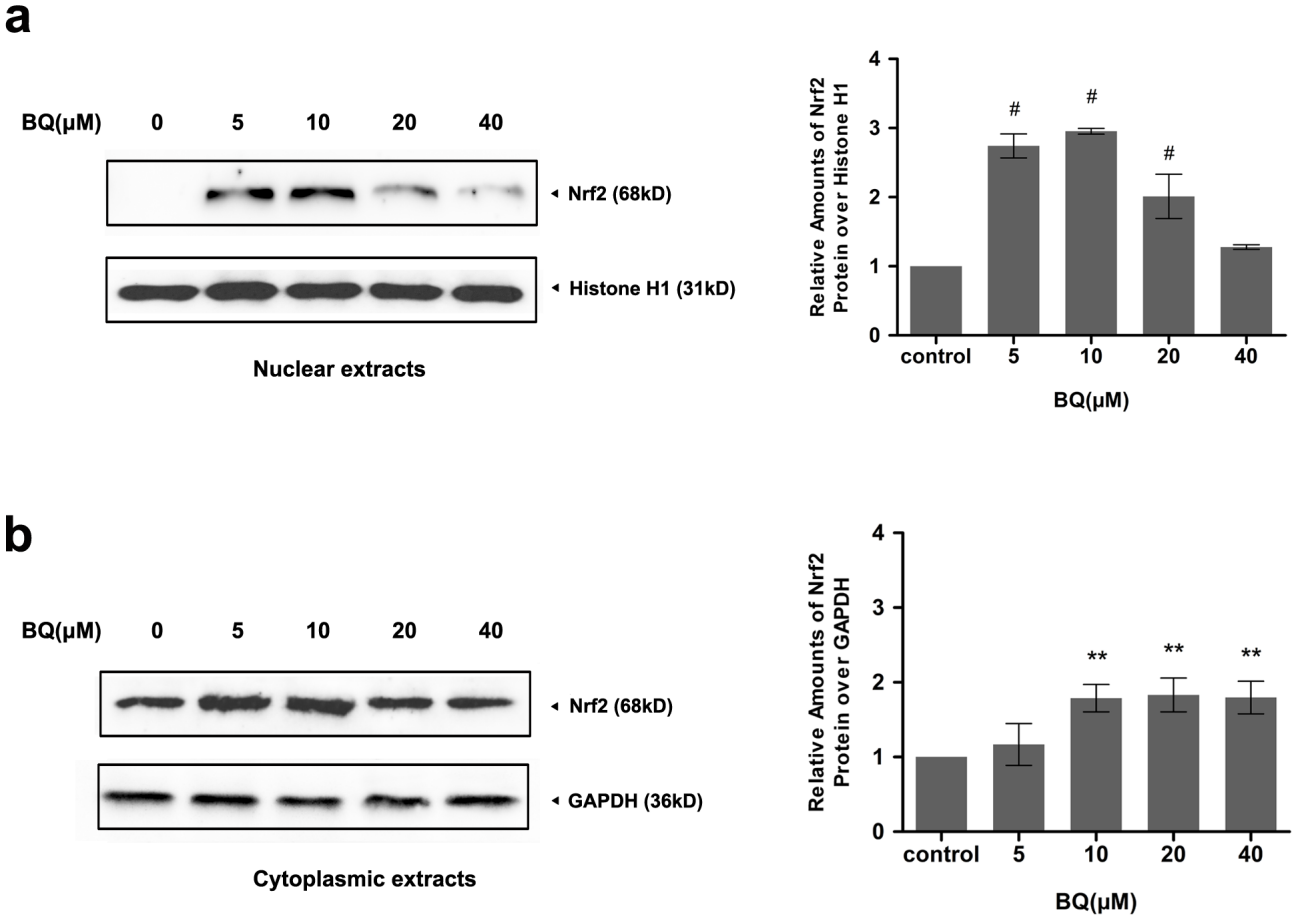
Exposure to BQ upregulated Nrf2 protein expression and caused Nrf2 nuclear accumulation. Western Blot analysis of nuclear and cytoplasmic extracts from YS-HSCs treated with increasing concentrations of BQ for 6 hours. Histone H1 and GAPDH antibodies were used as loading controls for nuclear and cytoplasmic extracts, respectively. Left panels: representative blotting results. Densitometry of the immunoblotting results was shown in the right panels. Values are presented as means ± SDs (n = 3−5). * *P*<0.05, **P<0.01, # *P*<0.001.

As the fact that intracellular ROS metabolism is predominantly regulated by Nrf2, changes in Nrf2 protein expression in response to BQ were examined in YS-HSCs. Because Nrf2 nuclear accumulation is a marker of Nrf2 activation, nuclear and cytoplasmic extracts were prepared to detect Nrf2 protein expression. As shown in Figure 5, a significant elevation of Nrf2 protein expression were observed in both nuclear and cytoplasmic extracts collected from the cells treated with various concentrations of BQ for 6 hours. These results suggest that exposure to BQ promoted Nrf2 nuclear accumulation and activation in YS-HSCs.

### Activation of Nrf2-ARE pathway in the BQ-treated YS-HSCs

**Figure 6:**
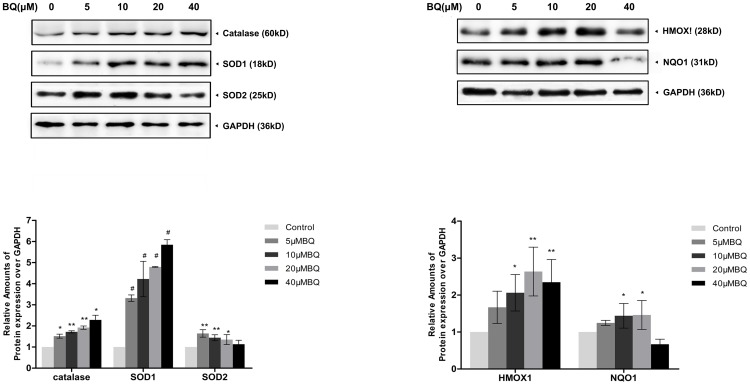
Increased expression of Nrf2-ARE pathway proteins were observed in the BQ-treated YS-HSCs. Induction of catalase, SOD1, SOD2, HMOX1 and NQO1 protein levels by BQ. Immunoblotting data were shown in the upper panel. GAPDH was measured as loading control. Densitometry of immunoblot results was shown in the lower panel. Values are presented as means ± SDs (n = 3−5). *, *P*<0.05; **, *P*<0.01; #, *P*<0.001 compared with controls.

To further investigate the effect of BQ on Nrf2 antioxidant program, the expression of various target proteins involved in the Nrf2-ARE pathway, including detoxification enzymes (NQO1) and antioxidant enzymes (catalase, SOD, and HO-1), were examined by Western Blotting. Increased expression of Nrf2 target proteins were observed following 6 hours BQ treatment (Figure 6). These data demonstrated BQ treatment activates the Nrf2-ARE pathway in the YS-HSCs.

### ROS contribute to the BQ-induced PKM2 degradation

**Figure 7:**
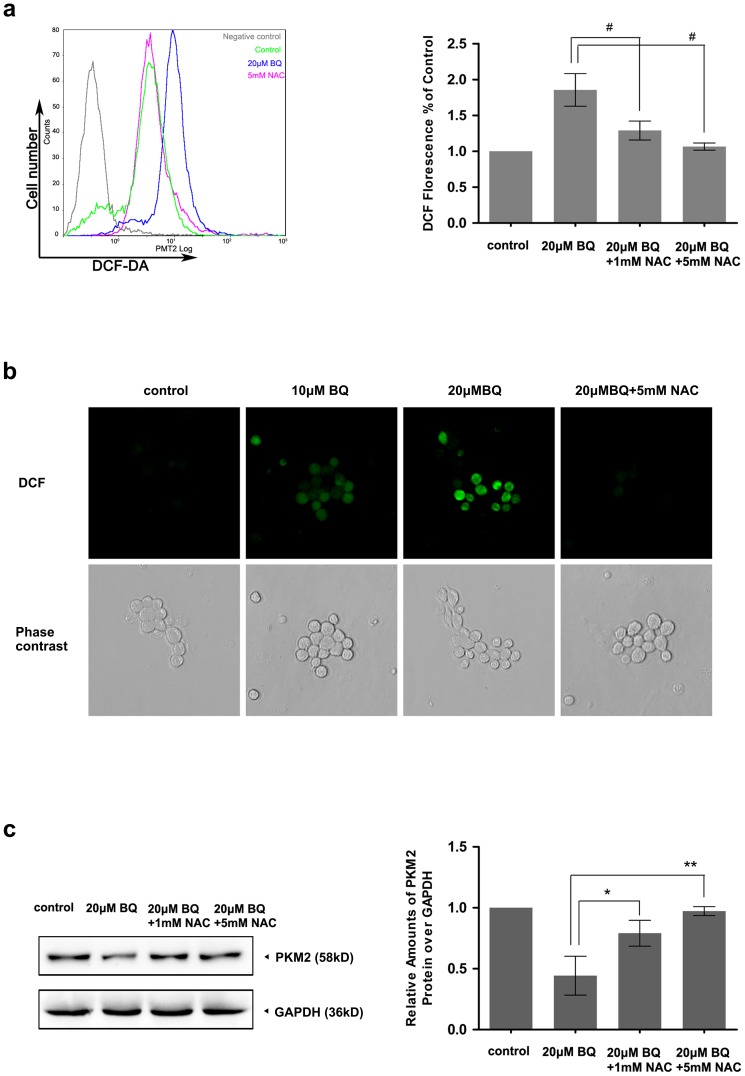
Role of ROS in BQ-induced PKM2 degradation. YS-HSCs were treated with 1 mM or 5 mM NAC for 2 hours, then exposed to 10 µM and 20 µM concentrations of BQ. NAC pretreatment decreased ROS generation caused by BQ (a) & (b). (a) The cells were analyzed by flow cytometry after treatment with 20 µM DCF-DA for 45 min. Illustrated overlays on the left panel reflected the ROS generation. Quantifications of ROS generation are displayed on the right pannel. (b) The cells were imaged by fluorescence microscopy after DCF-DA staining. Representative images selected from randomly chosen fields are shown. (c) Scavenging of ROS prevented PKM2 protein degradation caused by BQ. The expression of PKM2 protein was analysed by western blotting. Immunoblotting of PKM2 was shown in the left panel. GAPDH was measured as loading control. Densitometry of immunoblot results was shown in the right panel. Values are presented as means ± SDs (n = 3−5). *, *P*<0.05; **P<0.01; #, *P*<0.001 represent significant differences between the experiments.

To test whether ROS generation is relevant for PKM2 degradation induced by BQ, we measured the effects of ROS inhibition on PKM2 protein expression levels. YS-HSC cells were treated with 20 µM BQ for 6 hours with or without pretreatment of the cells with N-acetylcysteine (NAC), a free radical scavenger, for 2 hours. As expected, 20 µM BQ treatment enhanced DCF staining substantially. Pretreatment of YS-HSC cells with the antioxidant NAC almost completely suppressed DCF staining (Figure 7a & 7b). Thus, pretreatment of NAC inhibited BQ-induced ROS generation. Notably, the degradation of PKM2 protein was almost completely inhibited by 5 mM NAC pretreatment (Figure 7c). These results confirmed that generation of ROS stimulated by BQ is required for BQ-induced PKM2 degradation.

ROS are important molecules in normal cell signaling. However, excessive generation of ROS can affect the intracellular redox states, leading to mutagenic changes and thereby promote oncogenesis. Cellular ROS are generated from various biological reactions. In the present study, the expression of NOX1 protein was induced by BQ exposure, accompanied by an increased ROS generation. These data indicated that NADPH oxidase is the major source of ROS in the cellular response to BQ.

**Figure 8:**
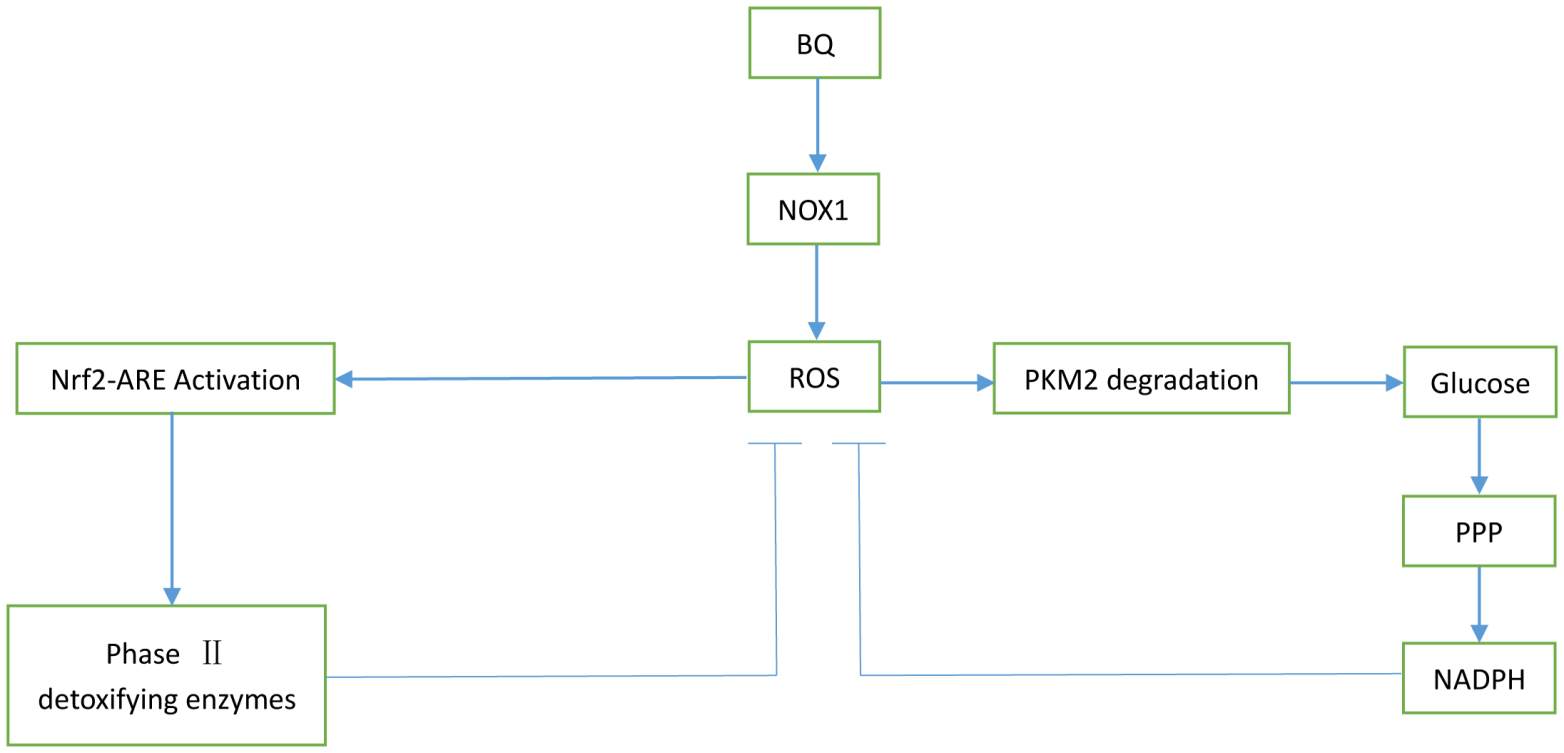
BQ induced ROS and two possible pathways involved in the ROS detoxification. Exposure to BQ causes NOX1 up-regulation, resulting in ROS generation in YS-HSCs. Intracellular ROS elevation promotes two possible pathways: (a) Nrf2 nuclear accumulation and activation of Nrf2-ARE pathways, including detoxification enzymes and antioxidant enzymes; (b) PKM2 degradation and promoting glucose flux into the pentose phosphate pathway (PPP), leading to generate NADPH responsible for the maintain of reducing glutathione, which plays a critical role in detoxification of ROS.

We had characterized the primary regulatory molecules and cell signaling pathway of antioxidant system. BQ exposure induced Nrf2 nuclear accumulated and activated the downstream proteins of Nrf2-ARE signaling pathways. Activation of Nrf2-ARE signaling pathways may play an important role in regulating the cellular redox state (Figure 8).

Cancer cells take up more glucose and produce increasable lactate regardless of the concentration of oxygen, this phenomenon is called Warburg effect [25]. PKM2 is a key enzyme in the glycolytic pathway and is found in all proliferative tissue cells, including tissue stem cells, in particular hematopoietic stem cells, and glycolytic metabolism also present in these cells [26]. Inactivation of PKM2 promote glucose metabolism and generate NADPH, to maintain glutathione in the reduced state, which plays a critical role in detoxification of ROS and oxidative stress damage repair process [27], [28] (Figure 8). . We observed the PKM2 protein expression was inhibited in response to BQ in YS-HSCs. The effect of BQ in reducing PKM2 expression may be a new mechanism that mediates the toxicity or carcinogenicity of the ROS in YS-HSCs. Pretreatment of antioxidant reduced PKM2 degradation, suggesting that the production of ROS by BQ contributes to BQ-induced PKM2 degradation. However, the mechanism by which ROS induced PKM2 degradation is still unknown. In human lung cancer cells, high levels of ROS cause inactivation of PKM2 by oxidation of Cys358 [23]. Whether oxidation of PKM2 involved in the BQ-induced PKM2 degradation in YS-HSCs still need further experimental verification.

## Supporting Information

Table S1 **Expression of hematopoietic surface makers in E8-10d yolk sac cells.**
(XLS)
